# Western Experience of Hepatolithiasis: Clinical Insights from a Case Series in a Tertiary Center

**DOI:** 10.3390/medicina61050860

**Published:** 2025-05-07

**Authors:** Natale Calomino, Ludovico Carbone, Engjell Kelmendi, Stefania Angela Piccioni, Gianmario Edoardo Poto, Giulio Bagnacci, Luca Resca, Annalisa Guarracino, Sergio Tripodi, Bina Barbato, Stefano Brillanti, Franco Roviello, Gian Luigi Adani, Daniele Marrelli

**Affiliations:** 1 Unit of Kidney Transplant, Department of Surgery, Azienda Universitaria Ospedaliera Senese, 53100 Siena, Italy; gianluigi.adani@ao-siena.toscana.it; 2 Unit of General Surgery, Department of Medicine Surgery and Neuroscience, University of Siena, 53100 Siena, Italy; l.carbone2@student.unisi.it (L.C.); e.kelmendi@student.unisi.it (E.K.); g.poto@student.unisi.it (G.E.P.); lucaresca3@gmail.com (L.R.); annalisa.guarraci@student.unisi.it (A.G.); franco.roviello@unisi.it (F.R.); daniele.marrelli@ao-siena.toscana.it (D.M.); 3 Unit of Surgical Oncology, Department of Oncology, Azienda Universitaria Ospedaliera Senese, 53100 Siena, Italy; stefania.piccioni@ao-siena.toscana.it; 4 Unit of Diagnostic Imaging, Department of Medicine Surgery and Neuroscience, University of Siena, 53100 Siena, Italy; giulio.bagnacci@unisi.it; 5 Section of Pathology, Department of Medical Biotechnology, University of Siena, 53100 Siena, Italy; s.tripodi@ao-siena.toscana.it (S.T.); bina.barbato@student.unisi.it (B.B.); 6 Unit of Gastroenterology, Department of Medicine Surgery and Neuroscience, University of Siena, 53100 Siena, Italy; stefano.brillanti@ao-siena.toscana.it

**Keywords:** hepatolithiasis, intrahepatic, bile duct stone, magnetic resonance cholangiopancreatography, surgery, hepatectomy, Western, case series

## Abstract

*Background and Objectives*: Hepatolithiasis (HL), or intrahepatic bile duct stone disease, shows regional variation and is a rare condition in Western countries. While cases from East Asia are often linked to chronic biliary infections and brown pigment stones, Western HL more frequently involves cholesterol or black pigment stones, typically in the context of prior cholecystectomy, biliary interventions, or congenital anomalies. The disease is generally associated with significant morbidity, including recurrent cholangitis, biliary strictures, and risk of cholangiocarcinoma. This study aimed to characterize HL disease in an Italian case series. *Materials and Methods*: We retrospectively reviewed 1450 patients with biliary stone disease treated between 2010 and 2024. HL was diagnosed in 14 patients (0.96%). Clinical records, imaging (ultrasound, CT, magnetic resonance cholangiopancreatography—MRCP, cholangiography), bile cultures, and stone composition (categorized as cholesterol, brown pigment, black pigment, or mixed using FTIR/XRD) were analyzed. *Results*: Among the 14 patients (mean age: 60.1 years; 64.3% female), 71.4% presented with recurrent cholangitis, while 28.6% were asymptomatic. Stones were left-sided in 57.1%, right-sided in 21.4%, and bilateral in 21.4%. Stone composition was cholesterol/mixed in 50%, brown pigment in 35.7%, and black pigment in 14.3%. Risk factors for bile stasis were present in 71.4% of cases. Bile cultures (available in nine cases) were positive in 77.8%. MRCP was highly effective for diagnosis. Hepatectomy achieved complete resolution in 35.7% of patients with unilobar disease; endoscopic/percutaneous therapy had a 44.4% recurrence rate. Interestingly, no cholangiocarcinoma was observed over a median follow-up of 4.8 years. *Conclusions*: Western HL is a rare, heterogeneous disease with distinct features. Cholesterol-predominant, infection-negative cases suggest a metabolic or surgical etiology. Hepatectomy offers durable outcomes in unilobar disease. Advanced imaging (MRCP, cholangioscopy) and personalized strategies are key to effective management.

## 1. Introduction

Hepatolithiasis (HL), defined as the presence of calculi within the intrahepatic bile ducts proximal to the confluence of the hepatic ducts, represents a rare but clinically significant hepatobiliary disorder in Western populations. The disease has long been endemic in East Asia, particularly in rural areas of China, Korea, Taiwan, and Japan, where it accounts for up to 20–30% of all biliary tract interventions and is commonly associated with recurrent pyogenic cholangitis, parasitic infections, and malnutrition-induced bile stasis [1,2,3,4]. Due to this geographic predilection, the condition has historically been termed “Oriental cholangiohepatitis”. However, with improvements in public sanitation and dietary standards, the incidence of HL has declined in Asia [5,6]. Conversely, Western countries have seen a modest increase in HL diagnoses, attributable to enhanced imaging modalities, greater clinical recognition, and increased migration from endemic regions [7,8].

The pathogenesis of HL is multifactorial. In endemic Eastern settings, HL is most often linked to chronic biliary infection and bile stasis secondary to environmental exposures such as Clonorchis sinensis or Opisthorchis viverrini infestation, low-protein diets, and poor hygiene. These conditions foster colonization by β-glucuronidase-producing bacteria that deconjugate bilirubin, leading to calcium bilirubinate precipitation and the formation of brown pigment stones [9,10,11]. In contrast, HL in Western cohorts is generally non-infectious and may be secondary to gallstone migration, post-surgical biliary strictures, congenital anomalies (e.g., Caroli disease), or metabolic predispositions such as ABCB4 (MDR3) mutations that impair phospholipid secretion [12,13,14]. The predominance of cholesterol or mixed stones and the frequent absence of biliary pathogens in Western HL support a distinct, non-infectious pathophysiology [15,16].

Although comprising less than 1% of hepatobiliary stone diseases in Western surgical series [17], HL poses unique diagnostic and therapeutic challenges due to its heterogeneous manifestations and risk of major complications, including recurrent cholangitis, hepatic abscesses, secondary biliary cirrhosis, and intrahepatic cholangiocarcinoma [18,19,20]. The disease course is often characterized by cycles of ascending infection and progressive ductal fibrosis. Recent advances in imaging, particularly magnetic resonance cholangiopancreatography (MRCP) and peroral cholangioscopy, have enhanced the detection and management of intrahepatic calculi and ductal anomalies, contributing to earlier diagnosis and improved therapeutic targeting [21,22,23].

However, there is a paucity of studies from Western countries addressing the clinical spectrum, microbiology, stone composition, and outcomes of HL. Existing classification systems are derived from Eastern cohorts and may not adequately reflect the disease profile observed worldwide [24,25,26]. In response to the clinical need to reassess the epidemiology, diagnostic frameworks, and therapeutic strategies, we conducted a case series of patients treated for biliary stone disease at a single tertiary referral center in Italy over a 14-year period.

This study aimed to characterize the clinical, radiological, microbiological, and pathological features of HL in an Italian case series and to compare these findings with established Eastern paradigms. Our objective was to highlight pathophysiological and clinical divergences, and to propose considerations for a refined classification system that could be applicable in a wider Western scenario.

## 2. Materials and Methods

Observational case series: A total of 1450 patients were treated for biliary stone disease at a single tertiary referral center in Italy between January 2010 and January 2024. Among these, 14 patients (0.96%) met the diagnostic criteria for HL, defined as the presence of calculi within the intrahepatic bile ducts proximal to the confluence of the right and left hepatic ducts.

Patients with stones confined solely to the gallbladder or extrahepatic bile ducts, without intrahepatic involvement, were excluded from the analysis. The diagnosis of HL was established through a combination of intraoperative findings, imaging modalities (including ultrasonography, computed tomography—CT, and MRCP), and endoscopic evaluations. Cases of primary sclerosing cholangitis were excluded.

Demographics and clinical history (including prior biliary surgeries, known biliary anomalies, and presenting symptoms), imaging findings (including, when applicable, endoscopic retrograde cholangiopancreatography [ERCP] or percutaneous transhepatic cholangiography [PTC]), microbiological analysis (bile samples obtained during surgery, endoscopy, or drainage), stone analysis (classified as brown pigment, black pigment, cholesterol, or mixed types), histopathology (chronic cholangitis, ductal fibrosis, epithelial dysplasia, or malignancy) were extracted from electronic health records.

Treatment was individualized and included endoscopic, percutaneous, or surgical options based on disease distribution, strictures, atrophy, and patient comorbidities. Procedures included hepatectomy, endoscopic sphincterotomy with stone extraction, SpyGlass-assisted cholangioscopy, and percutaneous lithotripsy.

Patients were followed longitudinally through clinic visits and imaging studies. Outcomes assessed included recurrence of stones or cholangitis, need for re-intervention, and development of intrahepatic cholangiocarcinoma. Median follow-up was 4.8 years (range 1.2–13.6 years).

### 2.1. Patient Stratification

The 14 HL cases were stratified into the following subgroups for comparative analysis: (1) stone composition: cholesterol, pigment (brown or black), or mixed; (2) presence or absence of biliary infection, as confirmed by positive bile cultures; (3) treatment modality: surgical (hepatectomy), endoscopic, percutaneous, or combined; (4) distribution of disease: unilobar (right or left) versus bilateral; and (5) predisposing factors: including prior cholecystectomy, biliary-enteric anastomosis, congenital anomalies, or suspected metabolic predisposition (e.g., ABCB4/MDR3 mutation).

Comparative analyses were conducted between groups with respect to clinical presentation, microbiological profiles, recurrence rates, and long-term outcomes.

### 2.2. Statistical Analysis

Given the small sample size and the descriptive nature of the study, statistical analyses were primarily exploratory. Median with interquartile ranges was used for continuous variables. Categorical variables were summarized as frequencies and percentages. Data analysis was performed using SPSS Statistics, version 27.0 (IBM Corp., Armonk, NY, USA).

This case series has been reported in line with the Preferred Reporting Of Case Series in Surgery (PROCESS) guideline.

## 3. Results

Fourteen consecutive patients were diagnosed with HL, confirming its rarity in Western clinical practice. The mean age at diagnosis was 61 years (range 52–71), with a predominance of female patients (nine females, five males; 64.3% vs. 35.7%) (Table 1).

Hypertension was the most frequent comorbidity, affecting approximately 50% of patients. Diabetes mellitus was present in 14.3% of patients, both cases associated with concurrent hypertension. Previous ischemic heart disease was documented in two patients.

### 3.1. Clinical Presentation and Risk Factors

Ten patients (71.4%) presented with clinical features consistent with recurrent cholangitis, characterized by right upper quadrant pain, fever, and jaundice. In contrast, four cases (28.6%) were diagnosed incidentally through imaging performed for unrelated clinical concerns or as part of post-cholecystectomy surveillance. Laboratory investigations at presentation frequently demonstrated elevated serum alkaline phosphatase and γ-glutamyl transpeptidase levels, leukocytosis, and mild hyperbilirubinemia.

Established risk factors for intrahepatic stone formation were identified in the majority of patients. A history of cholecystectomy or gallbladder calculi, suggestive of secondary stone migration, was present in six individuals (42.9%). Biliary strictures of either iatrogenic or inflammatory origin were noted in three patients (21.4%). One patient (7.1%) was diagnosed with segmental Caroli disease, a congenital disorder associated with impaired bile drainage. Notably, two patients (14.3%) were East Asian immigrants with documented prior infection with Clonorchis sinensis, consistent with the classical infectious phenotype observed in endemic regions.

### 3.2. Figures, Tables, and Schemes

**Figure 1:**
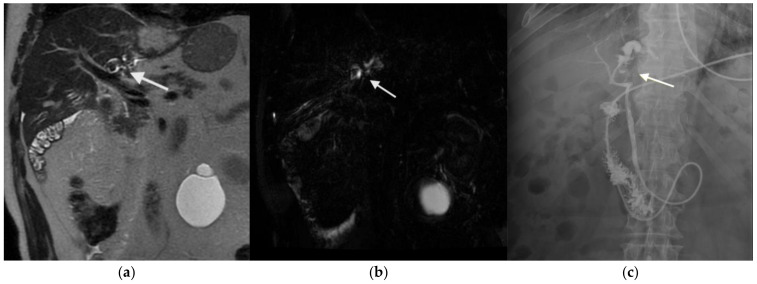
(**a**,**b**) Magnetic resonance imaging of a patient with suspected intrabiliary lithiasis, shown in T2-weighted FSE and MRCP sequences. A round, hypointense structure is visible in the proximal segment of the left main biliary duct, consistent with a biliary stone; (**c**) ERCP a few days after demonstrating the calculus as a filling defect surrounded by contrast medium.

All patients underwent US as the initial imaging modality. This revealed intrahepatic ductal dilatation in 11 cases (78.6%) and echogenic foci suggestive of calculi in 7 cases (50%). Cross-sectional imaging with CT scan was performed in nine patients, with intrahepatic calcifications or ductal abnormalities identified in six cases. MRCP was conducted in 12 patients (85.7%), offering high-resolution assessment of stone burden, biliary anatomy, and associated strictures. MRCP demonstrated a sensitivity of 100% for the detection of intrahepatic stones when compared to intraoperative findings (Figure 1).

**Figure 2:**
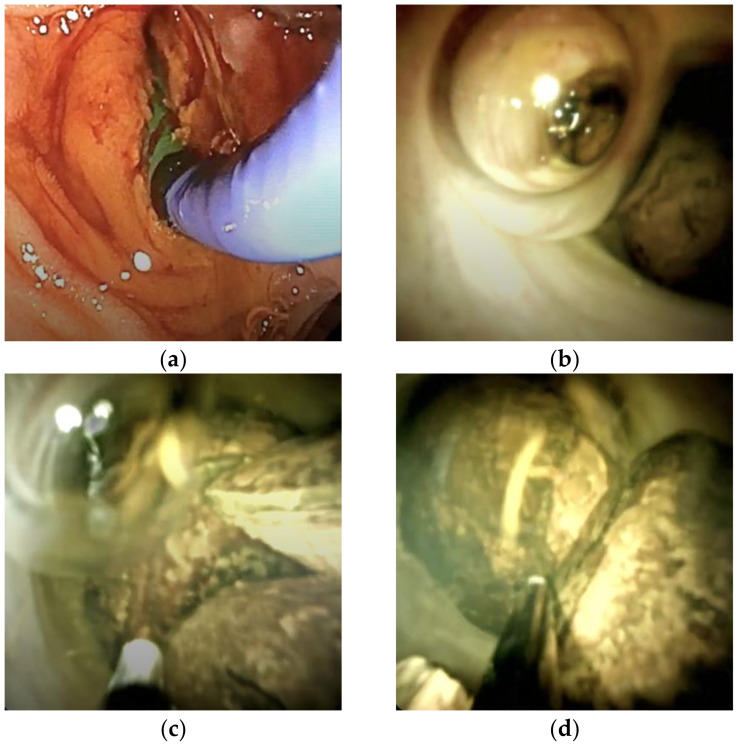

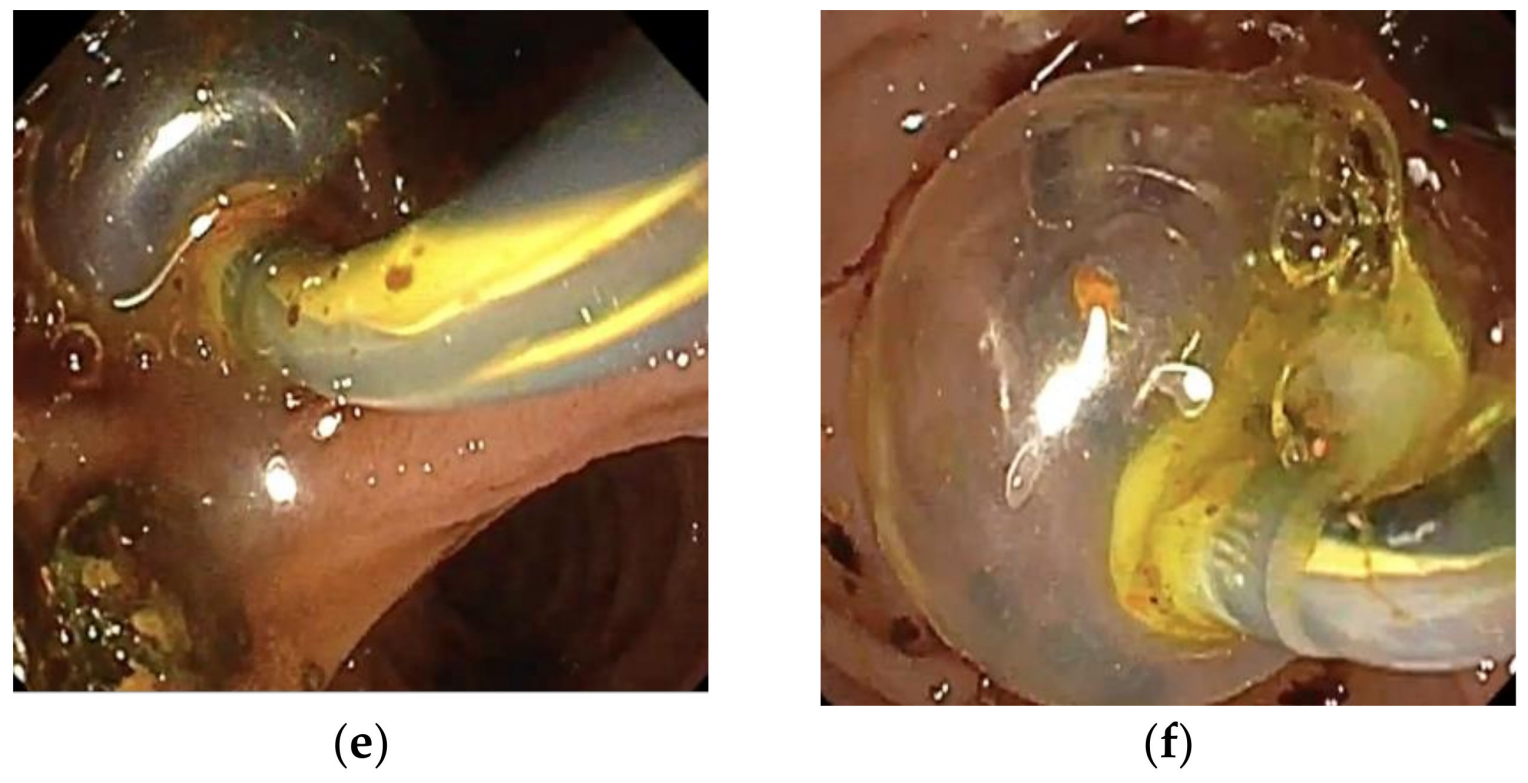
(**a**,**b**) Assessing multiple large stones; (**c**,**d**) fragmenting a stone; (**e**,**f**) removing by extraction balloon using the SpyGlass system.

In three complex cases, peroral digital cholangioscopy utilizing the SpyGlass system after ERCP was employed. This technique enabled direct visualization and facilitated fragmentation of impacted stones located within strictured biliary segments (Figure 2).

Overall, intrahepatic stones were localized to the left hepatic lobe in eight patients (57.1%), the right lobe in three patients (21.4%), and were bilobar in distribution in the remaining three patients (21.4%). Concomitant biliary ductal abnormalities were identified in 10 cases (71.4%), comprising 7 strictures, 3 instances of segmental hepatic atrophy, and 6 cases of ductal dilatation.

### 3.3. Stone Composition

**Figure 3:**
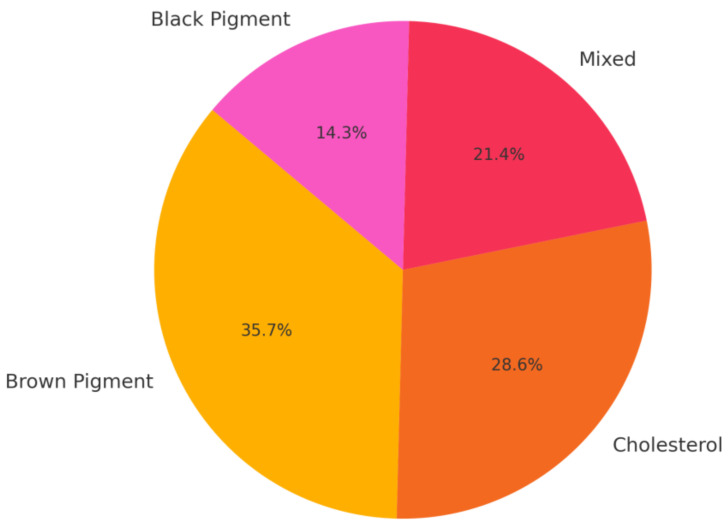
Stone composition in our case series affected by hepatolithiasis.

After surgery, the composition of the intrahepatic stones was analyzed and classified as follows (Figure 3): cholesterol stones were identified in four patients (28.6%), brown pigment stones in five patients (35.7%) and black pigment stones in two patients (14.3%). Mixed stones, containing both cholesterol and pigment components, were observed in three patients (21.4%).

Cholesterol and mixed stones were more frequently observed in younger patients and those without active infection or prior biliary surgery. In contrast, all patients with brown pigment stones had positive bile cultures and clinical or histological evidence of chronic cholangitis.

### 3.4. Microbiological Findings

Bile cultures were obtained from nine patients. Of these cultures, seven (77.8%) gave positive results, mainly from enteric organisms: Escherichia coli in five patients, Enterococcus faecalis in three patients, Klebsiella pneumoniae in two patients, and mixed anaerobes in three patients. Three patients had polymicrobial growth and all culture-positive cases were brown pigment stone patients. The cholesterol stone cases had sterile bile samples.

### 3.5. Treatment Outcomes

**Figure 4:**
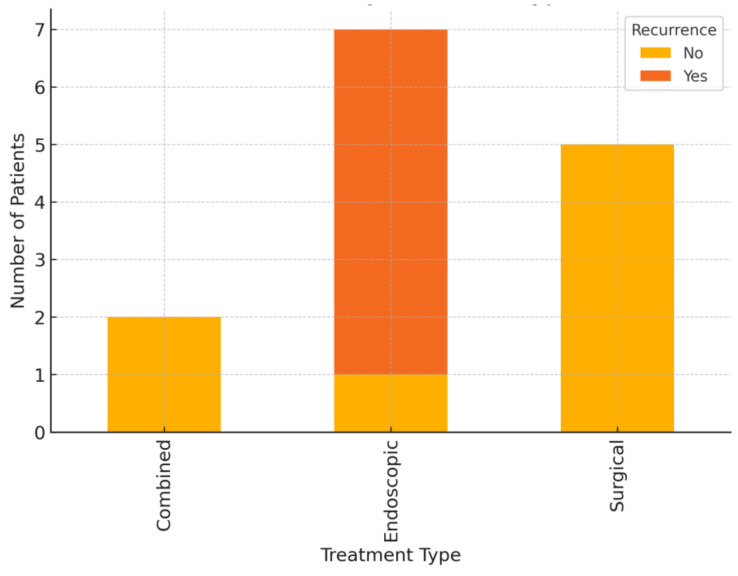
Recurrence of hepatolithiasis disease by treatment type.

Management strategies included surgical resection, endoscopic intervention, and combined modalities as shown in Figure 4.

Surgical resection was performed in five patients (35.7%) with unilobar disease accompanied by segmental atrophy or strictures. Four patients underwent left lateral sectionectomy (segments II and III), while one patient underwent a right hepatectomy. Complete stone clearance was achieved in all cases, with no evidence of recurrence during the follow-up period.

Endoscopic therapy was employed in seven patients (50%) and consisted of ERCP with sphincterotomy and stone extraction. In three cases, cholangioscopy-assisted lithotripsy was required due to impacted stones or stones located within intrahepatic branches.

Percutaneous transhepatic cholangioscopy and lithotripsy were performed in one case with challenging biliary access and segmental strictures.

Combined approaches were utilized in two patients with bilobar disease, involving surgical resection of the more severely affected hepatic lobe, followed by endoscopic clearance of residual stones in the contralateral lobe.

No perioperative mortalities were reported. A single surgical case was complicated by transient biliary leakage, which was managed conservatively.

## 4. Discussion

Our group of patients showed a low incidence of the disease (less than 1% of biliary stone patients) and an etiologic profile distinct from those reported in Eastern studies [5]. As in other Western reports, most cases involved secondary intrahepatic stones, either retained or migrated gallstones, or developed after biliary interventions, strictures, or congenital anomalies [27]. In contrast to East Asia, where primary HL is predominantly infection-related and associated with brown pigment stones, our patient sample exhibited a higher prevalence of cholesterol stones in sterile bile compared to Asian populations (50%), suggesting underlying metabolic or anatomical etiologies. For instance, one patient with segmental Caroli’s disease developed large cholesterol stones without infection in an isolated hepatic segment, similar to low-phospholipid-associated cholelithiasis (linked to MDR3 gene mutation) reported in other series [28]. These findings underscore that Western disease is commonly driven by chronic cholestasis (e.g., due to strictures or sphincter of Oddi dysfunction) and metabolic alterations in bile [29], while HL more often originates from infection and biliary parasites in East Asia [30].

Our analysis reinforces the central role of the biliary microbiome. Although many stones were initially sterile, secondary infections can arise once obstruction occurs. Bile cultures were positive in 78% of tested patients, often yielding enteric organisms (E. coli, Klebsiella, Enterococcus). These bacteria produce β-glucuronidase, which deconjugates bilirubin to form insoluble calcium bilirubinate, which is a key step in pigment stone formation [31]. In our group, all patients with brown pigment stones had positive bile cultures and chronic cholangitis, consistent with infection-driven pathogenesis. Conversely, cholesterol stone patients typically had negative cultures, suggesting a predominantly metabolic or immunologic mechanism. Nevertheless, once strictures and stones are present, patients may enter a cycle of stasis, infection, and further ductal damage [32]. This can progress to secondary biliary cirrhosis (14% of cases) or cholangiocarcinoma (1.3 to 23% risk). No malignancies were observed in our series, but longer-term surveillance is warranted.

Another central role is played by advanced imaging [33]. MRCP primarily identified intrahepatic stones, including non-calcified cholesterol stones that may be missed by CT scan [34], and delineated ductal strictures with over 95% sensitivity. Peroral cholangioscopy allowed direct visualization of the biliary tree and targeted clearance, especially in strictured ducts [35]. These techniques replaced older approaches like exploratory surgery or T-tube cholangiograpy [36]. One patient underwent EUS-guided hepaticogastrostomy for a left duct stone inaccessible by ERCP, highlighting the utility of newer endoscopic strategies. A multimodal imaging approach in our group of patients (US, MRCP, ERCP/cholangioscopy, EUS) optimized management and improved diagnostic accuracy, including malignancy exclusion via cholangioscopic biopsy [37,38].

Surgical treatment was effective in localized disease. All five patients with unilobar HL underwent anatomical resection (typically left lateral segmentectomy for left-sided stones) with minimal recurrence [39]. Our surgical outcomes matched with those from Asia, where 90–95% complete clearance is reported with hepatectomy, compared to 60–70% with endoscopic clearance alone. Bilateral stones present a therapeutic challenge. For such cases, we adopted resection of an atrophic lobe with dense stones while clearing the contralateral ducts endoscopically to avoid residual stone burden. ERCP with sphincterotomy and balloon extraction was the first-line approach, especially in non-strictured cases [40]. Although most patients achieved clearance, multiple sessions were sometimes needed. Liver transplantation was not required, but it remains an option in diffuse, bilateral HL with liver failure or refractory cholangitis (Dong’s Type IIc classification). For milder disease or high-risk surgical candidates [41], we occasionally used conservative strategies such as antibiotics for cholangitis and long-term ursodeoxycholic acid (UDCA) in frail patients with asymptomatic cholesterol stones [42]. While UDCA improves bile flow, its efficacy in intrahepatic pigment stones remains unproven. Our practice emphasizes that, as in Eastern countries, a one-size-fits-all strategy is inadequate in Western settings. Treatment must be individualized based on stone distribution (localized vs. diffuse), stone type (infection-associated vs. metabolic), and patient-specific factors.

Long-term management requires ongoing surveillance even after apparent clearance. Even after stone clearance, relapses occurred due to residual stenosis. We recommend annual imaging (US or MRCP). Two patients developed cholangitis over 3 years after first clearance. While no malignancies were observed, cholangiocarcinoma remains a concern, particularly in patients with chronic cholangitis or recurrent disease. Surveillance may include bile cytology or cholangioscopic biopsies in high-risk cases. Post-cholecystectomy HL represents a unique Western phenotype. Routine imaging in patients with unexplained cholestatic enzymes may facilitate early detection.

Furthermore, our findings suggest the need to refine current classification systems for HL. No universally accepted classification exists, and prior systems vary in focus (anatomy, primary vs. secondary stones, or surgical strategy). A refined classification should integrate (1) anatomical distribution (uni- vs. bilateral, hilum involvement, strictures/atrophy), (2) stone composition (pigment–infection vs. cholesterol–metabolic), and (3) underlying etiologies (e.g., parasite exposure, prior biliary surgery, genetic predisposition). For example, sterile cholesterol stones in patients without biliary–enteric anastomosis may indicate a metabolic basis, warranting genetic and metabolic workup (e.g., MDR3, lipid profile). In contrast, infection-driven pigment stones often benefit from resection and drainage. Microbiological status and metabolic testing can guide stratification. This has therapeutic implications: infection-prone patients may benefit from antimicrobial or microbiome-targeted strategies; those with metabolic stones may respond to UDCA or bile flow modulation.

Certainly, given the lack of a control group and the small, potentially biased sample, case series are inherently limited in establishing causal relationships and generalizing findings to broader populations. We encourage the validation of our findings through larger studies conducted on broader Western cohorts.

## 5. Conclusions

In this single-center case series, HL emerged as a rare but clinically significant condition, comprising <1% of biliary stone cases. Western HL patients were typically older, predominantly female, and more likely to present with cholesterol-based or secondary stones following gallstone disease. This differs markedly from the infection-associated brown pigment stones described in Eastern cohorts. Our results underscore the value of advanced imaging (MRCP, cholangioscopy) in accurate diagnosis. Timely surgical intervention in localized disease yields superior outcomes. We argue for an updated classification of HL that goes beyond geographical distinctions and incorporates stone composition, microbiological status, and metabolic predisposition into the traditional anatomical framework.

## Figures and Tables

**Table 1:** Summary of our case series affected by hepatolithiasis.

Patient ID	Age	Sex	Stone Location	Stone Type	Bile Culture	Treatment	Recurrence	Follow-Up
P1	62	F	Left	Cholesterol	Negative	Surgical	No	5.0
P2	58	M	Left	Brown Pigment	Positive	Endoscopic	Yes	6.2
P3	45	F	Right	Cholesterol	Negative	Surgical	No	8.1
P4	74	F	Left	Mixed	Positive	Combined	No	4.2
4.7	69	F	Bilateral	Brown Pigment	Positive	Endoscopic	Yes	4.7
P6	55	M	Left	Black Pigment	Negative	Endoscopic	Yes	5.3
P7	60	F	Right	Brown Pigment	Positive	Endoscopic	Yes	2.1
P8	32	F	Left	Cholesterol	Negative	Surgical	No	7.6
P9	70	M	Bilateral	Mixed	Positive	Combined	No	1.8
P10	64	F	Right	Brown Pigment	Positive	Endoscopic	Yes	9.0
P11	48	M	Left	Cholesterol	Negative	Surgical	No	4.5
P12	79	F	Bilateral	Mixed	Positive	Endoscopic	No	2.9
P13	53	M	Left	Black Pigment	Negative	Endoscopic	Yes	3.8
P14	82	F	Left	Brown Pigment	Positive	Surgical	No	3.5

## Data Availability

Data supporting the reported results are available from the corresponding author upon reasonable request.

## Abbreviations

The following abbreviations are used in this manuscript:

HL	Hepatolithiasis
MRCP	Magnetic resonance cholangiopancreatography
CT	Computed tomography
ERCP	Endoscopic retrograde cholangiopancreatography
PTC	Percutaneous transhepatic cholangiography
EUS	Endoscopic ultrasound
UDCA	Ursodeoxycholic acid

## References

[1] Motta R.V., Saffioti F., Mavroeidis V.K. (2024). Hepatolithiasis: Epidemiology, presentation, classification and management of a complex disease. World J. Gastroenterol..

[2] De Oliveira1 R.S., da Silva P., Queiroz C.A.S., Terra-Júnior J.A., Crema E. (2018). Prevalence of bacteriobilia in patients undergoing elective colecystectomy. ABCD Arq. Bras. Cir. Dig..

[3] Tazuma S., Nakanuma Y. (2015). Clinical features of hepatolithiasis: Analyses of multicenter-based surveys in Japan. Lipids Health Dis..

[4] Zheng L., Ye Z.-Y., Ma J.-J. (2025). Effect of cholesterol metabolism on hepatolithiasis. World J. Gastroenterol..

[5] Shoda J. (2003). Hepatolithiasis-epidemiology and pathogenesis update. Front. Biosci..

[6] Sheen-Chen S.-M., Cheng Y.-F., Chen F.-C., Chou F.-F., Lee T.-Y. (1998). Ductal dilatation and stenting for residual hepatolithiasis: A promising treatment strategy. Gut.

[7] Dorrance H.R., Lingam M.K., Hair A., Oien K., O’Dwyer P.J. (1999). Acquired abnormalities of the biliary tract from chronic gallstone disease11No competing interests declared. J. Am. Coll. Surg..

[8] Sharma C.K. (2025). Significant Microbial Pathogenesis Perspective of Biliary Diseases. Infect. Disord. Drug Targets.

[9] Melichar B., Zezulová M. (2011). The significance of altered gastrointestinal permeability in cancer patients. Curr. Opin. Support. Palliat. Care.

[10] Li G., Gu R., Wen X., Wei D., Ming X., Chen H. (2012). The Effect of Early Enteral Nutrition on Hyperthermic Intraoperative Intraperitoneal Chemotherapy–Induced Mucosal Permeability Following Gastrectomy. J. Parenter. Enter. Nutr..

[11] Krentz T., Allen S. (2017). Bacterial translocation in critical illness. J. Small Anim. Pract..

[12] Nagpal R., Yadav H. (2017). Bacterial Translocation from the Gut to the Distant Organs: An Overview. Ann. Nutr. Metab..

[13] Kiecka A., Szczepanik M. (2023). Proton pump inhibitor-induced gut dysbiosis and immunomodulation: Current knowledge and potential restoration by probiotics. Pharmacol. Rep..

[14] Trauner M., Fickert P., Wagner M. (2007). MDR3 (ABCB4) Defects: A Paradigm for the Genetics of Adult Cholestatic Syndromes. Semin. Liver Dis..

[15] Roa J.C., García P., Kapoor V.K., Maithel S.K., Javle M., Koshiol J. (2022). Gallbladder cancer. Nat. Rev. Dis. Prim..

[16] Wang P., He Y., Ma X., Sun B., Huang B., Zhu C., Liu Y. (2015). Expression and Significance of COX-2 and Ki-67 in Hepatolithiasis with Bile Duct Carcinoma. Med. Sci. Monit..

[17] Vetrone G., Ercolani G., Grazi G.L., Ramacciato G., Ravaioli M., Cescon M., Varotti G., Del Gaudio M., Quintini C., Pinna A.D. (2006). Surgical Therapy for Hepatolithiasis: A Western Experience. J. Am. Coll. Surg..

[18] Chen M.-F., Jan Y.-Y., Jeng L.-B., Hwang T.-L., Wang C.-S., Chen S.-C., Chao T.-C., Chen H.-M., Lee W.-C., Yeh T.-S. (1999). Intrahepatic cholangiocarcinoma in Taiwan. J. Hepato-Biliary-Pancreatic Surg..

[19] Ohta T., Elnemr A., Yasui T., Kitagawa H., Kayahara M., Fushida S., Nishimura G.-I., Nagakawa T., Miwa K., Yamamoto M. (1999). Expression of nerve growth factor in hepatolithiasis. Liver.

[20] Ohta T., Nagakawa T., Konishi I., Ueno K., Kanno M., Akiyama T., Kayahara M., Izumi R., Konishi K., Miyazaki I. (1988). Clinical experience of intrahepatic cholangiocarcinoma associated with hepatolithiasis. JPN J. Surg..

[21] Ohta T., Nagakawa T., Ueda N., Nakamura T., Akiyama T., Ueno K., Miyazaki I. (1991). Mucosal dysplasia of the liver and the intraductal variant of peripheral cholangiocarcinoma in hepatholithiasis. Cancer.

[22] Feng L., Xia D., Yan L. (2016). Liver transplantation for hepatolithiasis: Is terminal hepatolithiasis suitable for liver transplantation?. Clin. Transpl..

[23] Shi W., Yang A.-M. (2021). Caroli disease: An update on pathogenesis. Chin. Med. J..

[24] Sakpal S.V., Babel N., Chamberlain R.S. (2009). Surgical management of hepatolithiasis. HPB.

[25] Calomino N., Scheiterle M.L.P.F., Fusario D., La Francesca N., Martellucci I., Marrelli D. (2021). Porcelain gallbladder and its relationship to cancer. Eur. Surg..

[26] D’Souza L.S., Bucobo J.C. (2020). Determining the Indeterminate in Biliary Strictures. Clin. Gastroenterol. Hepatol..

[27] Ananthakrishnan A.N., Saeian K. (2007). Caroli’s disease: Identification and treatment strategy. Curr. Gastroenterol. Rep..

[28] Leung J.W., Yu A.S. (1997). Hepatolithiasis and biliary parasites. Baillieres Clin. Gastroenterol..

[29] Bull L.N., Thompson R.J. (2018). Progressive Familial Intrahepatic Cholestasis. Clin. Liver Dis..

[30] Binda C., Gibiino G., Coluccio C., Sbrancia M., Dajti E., Sinagra E., Capurso G., Sambri V., Cucchetti A., Ercolani G. (2022). Biliary Diseases from the Microbiome Perspective: How Microorganisms Could Change the Approach to Benign and Malignant Diseases. Microorganisms.

[31] Sheen-Chen S.-M., Chen W.-J., Eng H.-L., Sheen C.-W., Chou F.-F., Cheng Y.-F., Lee T.-Y. (2000). Bacteriology and antimicrobial choice in hepatolithiasis. Am. J. Infect. Control.

[32] Ludwig D.R., Anderson M.A., Itani M., Sharbidre K.G., Lalwani N., Paspulati R.M. (2022). Secondary sclerosing cholangitis: Mimics of primary sclerosing cholangitis. Abdom. Radiol..

[33] Kim H.J. (2015). Hepatolithiasis and intrahepatic cholangiocarcinoma: A review. World J. Gastroenterol..

[34] Itani M., Lalwani N., Anderson M.A., Arif-Tiwari H., Paspulati R.M., Shetty A.S. (2021). Magnetic resonance cholangiopancreatography: Pitfalls in interpretation. Abdom. Radiol..

[35] Subhash A., Buxbaum J.L., Tabibian J.H. (2022). Peroral cholangioscopy: Update on the state-of-the-art. World J. Gastrointest. Endosc..

[36] Padmore G., Sutherland F.R., Ball C.G. (2021). The art and craft of biliary T-tube Use. J. Trauma. Acute Care Surg..

[37] Pizzicannella M., Boskoski I., Perretta S. (2020). Peroral Cholangioscopy: How Technology and Imaging Have Changed ERCP. J. Laparoendosc. Adv. Surg. Tech..

[38] Giovannini M. (2019). EUS-guided hepaticogastrostomy. Endosc. Ultrasound.

[39] Li R., Shan B., Tian K., Zhang X., Xie X. (2021). Biliary tract exploration via left hepatic duct stump versus the common bile duct incision in left-sided hepatolithiasis: A meta-analysis. ANZ J. Surg..

[40] Ishihara Y., Matsumoto K., Kato H., Tsutsumi K., Tomoda T., Matsumi A., Miyamoto K., Yamazaki T., Saragai Y., Fujii Y. (2021). Treatment outcomes, including risk factors of stone recurrence, for hepatolithiasis using balloon-assisted endoscopy in patients with hepaticojejunostomy (with video). Surg. Endosc..

[41] Feng X. (2012). Classification and management of hepatolithiasis: A high-volume, single-center’s experience. Intractable Rare Dis. Res..

[42] Ros E., Navarro S., Bru C., Gilabert R., Bianchi L., Bruguera M. (1993). Ursodeoxycholic acid treatment of primary hepatolithiasis in Caroli’s syndrome. Lancet.

